# Obscure gastrointestinal bleeding caused by congenital enteropathy in a Chinese young child-a case report

**DOI:** 10.1186/s12887-020-02333-0

**Published:** 2020-09-17

**Authors:** Youhong Fang, Weizhong Gu, Youyou Luo, Jie Chen

**Affiliations:** 1 Department of Gastroenterology, The Children’s Hospital, Zhejiang University School of Medicine, National Clinical Research Center for Child Health, 3333 Bin Sheng RoadZhejiang Province, Hangzhou, 310052 China; 2 Department of Pathology, The Children’s Hospital, Zhejiang University School of Medicine, National Clinical Research Center for Child Health, Hangzhou, China

**Keywords:** Anemia, *SLCO2A1*, CEAS, Obscure gastrointestinal bleeding, Small bowel ulcer

## Abstract

**Background:**

*SLCO2A1* was recently reported to cause nonspecific ulcers at small bowel, it was named as chronic enteropathy associated with *SLCO2A1* (CEAS). It was rarely reported beyond the Japanese population.

**Case presentation:**

A 4-year-5-month old girl presented with intractable anemia since 1-year-3-month. Her stool occult blood test was positive and the result of esophagogastroduodenoscopy and colonoscopy were normal. She was considered as obscure gastrointestinal bleeding. The magnetic resonance enterography and ultrasound of small intestinal revealed segmental thickening of small bowel. The capsule endoscopy detected ulcers, erosion and slightly stenosis near the site of junction of jejunum and ileum. She was considered chronic non-specific multiple ulcers of the small intestine and was advised to have whole exon sequencing. She was treated with exclusive enteral nutrition and iron supplement for two months. However, she was not responsive to this treatment, then she had three doses of infliximab. At the same time, the next-generation sequencing of this patient revealed two novel compound heterozygous mutations in *SLCO2A1*. She was diagnosed with CEAS and was treated with oral mercaptopurine. Her hemoglobin level was stable and the serum albumin level was slightly decreased during the follow up.

**Conclusion:**

CEAS may present as nonspecific small bowel ulcers, and misinterpret as small bowel Crohn’s disease. Genetic tests may help with the precise diagnosis of small bowel ulcers.

## Background

Obscure gastrointestinal bleeding is defined as bleeding of unknown origin that persists or recurs, after negative initial evaluation using upper and lower endoscopy and radiologic small bowel imaging [1]. The etiology of OGIB including inflammation, small vascular malformations, and polyps et al. Recently, a new gene *SLCO2A1* encodes a prostaglandin transporter was reported to cause small bowel nonspecific ulcers and sometimes cause stricture of small bowel which named as chronic enteropathy associated with *SLCO2A1* (CEAS). The main presentation of CEAS reported was anemia. To date, most of the cases reported about this disease were from Japanese population. Here we report a case of Chinese young girl who presented with chronic anemia due to OGIB and finally diagnosed as CEAS.

## Case presentation

A 4-year-5-month old girl was referred to our ward with complaint of anemia for more than three years. She was detected iron deficiency anemia at one year and three months old. She had no dizziness, no cough, no fatigue, no hematuria, no abdominal pain, no melena or bloody stool. Her appetite and normal nutritional status were normal. She was the first child in her family, and her parents and sister didn’t have anemia. Her blood routine test showed the hemoglobin level was 96 g/L, mean corpuscular volume (MCV) was 79.9 fL, mean corpuscular hemoglobin (MCH) was 24.9 pg, mean corpuscular hemoglobin concentration was (MCHC) 312 g/L, which indicated a hypochromic microcytic anemia. She was referred to the hematologist, and the results of etiology tests for anemia were showed in the Table 1. High resolution melting did not detect hot mutations of thalassemia and the bone marrow morphology test revealed hypochromic microcytic anemia. During the three years, she accepted iron supplementation intermittently. Her hemoglobin level was ranging from 67 to 120 g/L.

**Table 1:** The results of laboratory tests of the patient

Laboratory tests	Results
The percentage of reticulocytes	0.8%—7% (0.5–1.5%)
Serum iron level	2.9–9.6μml/L (8.9–32.3μml/L)
Serum ferritin level	2.0—6.2 μg/L (11-306μml/L)
Serum total iron binding capacity	48.5–64.7 μmol/L (54-77 μmol/L)
Glucose-6-phosphate dehydrogenase	44.8 U/dL (> 26U/dL)
Serum levels of vitamin B12	760 (180–914) pg/ml
Serum levels of folic acid	11.2 (3.1–20.5) ng/ml
Direct and indirect Coomb’s tests	Negative
Hemoglobin electrophoresis	Normal
Erythrocyte sedimentation rate	11 (0–20) mm/h
C-reactive protein	< 0.5 mg/L
Serum albumin	29.9–40.9 (32–52) g/L
Fecal calprotectin	> 1800 (< 200 ng/g)
Serum immunoglobin levels	
Ig G	5.6 (5–10.6) g/L
Ig M	1.31 (0.44–1.44) g/L
Ig A	1.12 (0.32–1.38) g/L
Ig E	< 18.9 (0–100) IU/ml
Subsets of T cells	
CD19	24.65 (21–33) %
CD3	61.60 (60–71) %
CD4/CD8	1.2:1 (1.9–2.9:1)
Antibodies of Epstein-barr virus	Negative
Antibodies of Cytomegalovirus	Negative
Interferon Gamma Release Assays	Negative
Antinuclear antibodies	Negative
Antineutrophil antibody	Negative

**Fig. 1:**
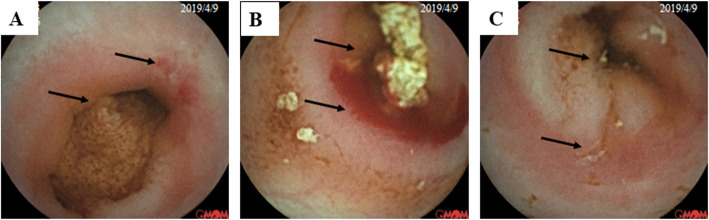
The capsule endoscopy revealed stricture, superficial ulcers and erosion in the small intestinal near the junction of jejunum and ileum

She was referred to a gastroenterologist because the stool occult blood test was positive sometimes. She had no history of taking aspirin or any other medicine. Her serum albumin level was 34 g/L. She was diagnosed with gastrointestinal bleeding, and started the initial laboratory tests (Table 1). The EGD and the colonoscopy didn’t detect ulcer or inflammation. The results of magnetic resonance enterography (MRE) and the ultrasound revealed segmental thickening of small intestinal wall, with the wall thickness of 0.46 cm and mainly involved the mucosal and submucosal of small intestinal. Capsule endoscopy didn’t reach the terminal ileum during the working time and a retention was observed. It revealed superficial ulcers and erosion at the location of stricture near the junction of jejunum and ileum (Fig. 1). She had double balloon endoscopy (DBE) through both mouth and anus. However, the DBE didn’t reach the location of stricture, and it did not reveal any other lesions. Based on the small bowel ulcers and stricture, anemia, mild hypoalbuminemia, and obviously increased fecal calprotectin, she was initially diagnosed as chronic non-specific multiple ulcers of the small intestine (CNSU) and very early onset small bowel intestinal Crohn’s disease was the main differential diagnosis. She was advised to have whole exome sequencing because of early disease onset and suspecting congenital disease. She was treated with exclusive enteral nutrition (EEN) and iron supplement after discussing with her parents.

She received EEN and iron supplement for eight weeks. The hemoglobin level was ranging between 90 and 107 g/L, and the repeated fecal calprotectin was still higher than 1800 ng/g. She received infliximab. However, she still had anemia and hypoalbuminemia after three doses of infliximab.

### Genetic analysis

**Fig. 2:**
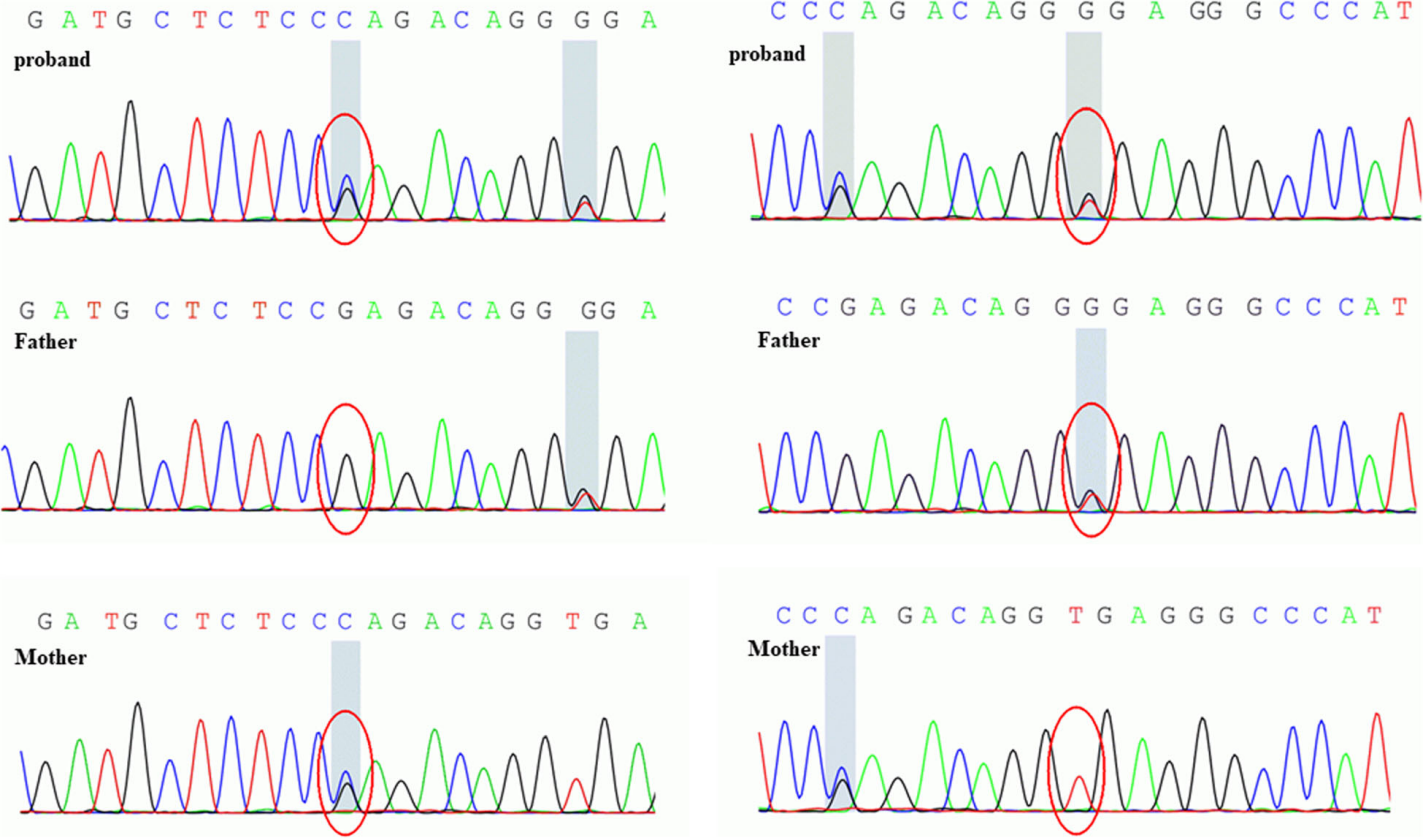
The next generation sequencing revealed novel compound mutations of *SLCO2A1*, the sanger sequencing confirmed the variants: chr3-133,654,624, c.1808G > C (p.R603P), chr3-133,654,616, c.1814 + 2 T > G, splice-5. The two mutations were derived from her mother and father separately

The next generation sequencing revealed two novel compound heterozygous mutations in *SLCO2A1*: chr3-133,654,624, c.1808G > C (p.R603P), chr3-133,654,616, c.1814 + 2 T > G (splice-5). The two variants were confirmed by sanger sequencing, and were derived from her mother and father, separately (Fig. 2). Her sister didn’t carry the variants.

### Immunohistochemical analysis of SLCO2A1 protein

**Fig. 3:**
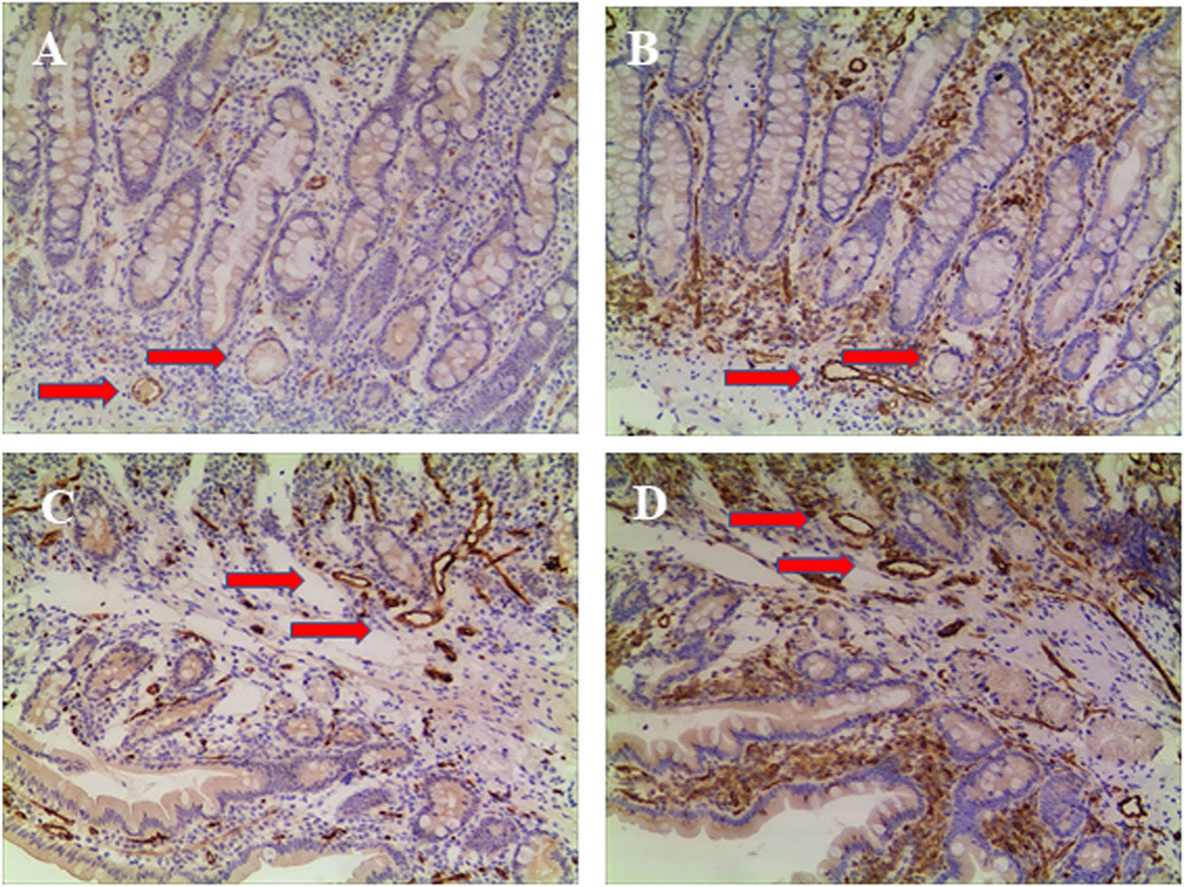
SLCO2A1 immunostaining. The duodenal tissue obtained by endoscopic biopsy from both of the patient (A × 100) and control (C × 100) were positive. CD31 immunostaining indicated the vascular endothelial cells of the capillary vessels in the mucosa of patient (B × 100) and control(D × 100)

SLCO2A1 expressions in the duodenal tissues were positive in patient and control (Fig. 3). The control with the complaint of intermitted abdominal pain for one year, and finally diagnosed with primary dysmenorrhea. CD31 immunostaining indicated the vascular endothelial cells of the capillary vessels in the mucosa of patient and control.

### Follow up

*SLCO2A1* mutation is related to the chronic nonspecific multiple ulcers of the small intestine. Combined with her history and character of her endoscopy findings, she was diagnosed as CEAS. There was no effective treatment according to the reported literatures about the treatment for CEAS. Some patients were responsive to the immunosuppressants, but were not responsive to the biological agents. She switched to mercaptopurine with the dose of 1 mg/kg per day. Up to the latest follow up, she didn’t have any abdominal symptoms and her hemoglobin level was increased from 76 g/L to 98 g/L, the serum albumin level was a bit decreased.

## Discussion and Conclusion

OGIB can be caused by small bowel ulcers, the ulcers can be interpreted as mucosal inflammation, vasculitis, drug-induced or nonspecific ulcers caused by inherited diseases. With the exploration to the last terminal of small intestinal by using of DBE and CE, increasing small intestinal diseases were recognized, among them there was a group of diseases presenting as nonspecific multiple ulcers in the small intestinal. It is identified with mutation of gene *SLCO2A1* and is also called CEAS [2].

We searched the keywords of “CEAS”, “nonspecific multiple ulcers” or “*SLCO2A1* and enteropathy” in the PUBMED and reviewed the literatures. So far as we know, all of the patients reported in the literatures diagnosed with CEAS were almost Japanese, except two patients recently reported by Korea [3] and Chinese [4]. Thus, we hypothesized that CEAS was underestimated or was not recognized in Chines patients. In this study, we reported a young child presented with OGIB caused by compound heterozygous mutations of *SLCO2A1*. This young child was mainly presented with anemia, and detected small bowel ulcers, erosion and stricture by capsule endoscopy.

A national study in Japan [5] including 65 CEAS patients identified 46 patients with *SLCO2A1* mutations at 11 sites. This disease predominantly occurs in woman, the male vs female ratio was 1:2.5, and it tends to affect the adolescents, with disease onset at 16.5 years (range, 1–69 years). There was only one literature focused on the child onset CEAS which reported four cases of CEAS children, and two patients were have disease onset within 2 years old [6]. The clinical features of these patients were mainly anemia 45 (98%), abdominal pain 18 (39%) and edema 11 (24%) [5]. CEAS ulcers are characterized by multiple, circular or eccentric oblique, shallow lesions with discrete margins [7]. In all the CEAS patients, the ileum is most frequently involved (98%-100%) [5], and the terminal ileum is not involved. While for small bowel Crohn’s disease, terminal ileum is the most common frequently involved. Patients with *SLCO2A1* mutation has negative immunohistochemical staining for SLCO2A1 while the patient reported here was positive. The immunohistochemical staining is useful in the diagnosis of CEAS. However, patients with deleterious mutations of *SLCO2A1* but not truncated mutation could not detect by immunohistochemical study [8]. Our patient may have the same condition.

There were several hot mutations reported in the Japanese population. Among the detected *SLCO2A1* mutations, a splicing mutation at intron 7 (c.940 + 1G > A; rs765249238) was the most frequently observed among CEAS patients, with 54% of mutation allele frequency [5]. Our patient had disease onset as early as one year and three months, and harbored two novel mutations, which were not reported in Japanese population. Mutation of c.1808 g > C, resulting amino acid changes from arginine to proline, the frequency of this mutation is 0.000024 among the normal population. Mutation of c.1814 + 2 T > G, causes splicing mutation at terminal 5 which resulting amino acid change. According to the American College of Medical Genetics and Genomics the variant of c.1808 g > C was identified as variants of unknown significance and the variant of c.1814 + 2 T > G was identified as Likely Pathogenic. The hot mutations in different genetic background maybe different.

*SLCO2A1* is also identified as a causal gene of primary hypertrophic osteoarthropathy (PHO) in 2012 [9]. Since then, many PHO patients were reported to have mutations of *SLCO2A1* [10–12]. Interestingly, among the PHO patients with *SLCO2A1* mutations over 50% percent patients had watery diarrhea and over 20% patients had anemia [13]. Unfortunately, there were no reports about the intestinal enteropathy in PHO patients. CEAS accompanied with PHO were previously reported [14–16]. Since *SLCO2A1* is shared with PHO, the major clinical manifestations of PHO, such as digital clubbing, periostosis, pachydermia were commonly observed in CEAS [5]. However, the PHO was more common observed in male while the CEAS was dominant in female. The relationship between CEAS and PHO are not clear. Our patient reported here didn’t show any typical features of PHO so far.

In conclusion, patients with nonspecific small intestinal ulcers with intractable anemia or hypoproteinemia should suspect CEAS. Although CEAS was rarely reported outside Japanese population, PHO was constantly reported. It may be under estimated in other population.

## Data Availability

There is no more case-specific data that could be shared.

## Abbreviations

CEAS: Chronic enteropathy associated with *SLCO2A1*
OGIB: Obscure gastrointestinal bleeding
EEN: Exclusive enteral nutrition
MRE: Magnetic resonance enterography
PHO: Primary hypertrophic osteoarthropathy
